# Significance of N6-Methyladenosine RNA Methylation Regulators in Immune Infiltrates of Ovarian Cancer

**DOI:** 10.3389/fgene.2021.671179

**Published:** 2021-07-07

**Authors:** Jing Gu, Fangfang Bi

**Affiliations:** Department of Obstetrics and Gynecology, ShengJing Hospital of China Medical University, Shenyang, China

**Keywords:** m6A RNA methylation regulators, immune cell infiltrates, immune checkpoint inhibitors, consensus clustering algorithm, ovarian cancer

## Abstract

N6-methyladenosine (m6A) RNA methylation regulators play an important role in the occurrence and development of tumors. Here, we aimed to identify the potential roles of m6A RNA methylation regulators in immune infiltrates of ovarian cancer. We obtained two distinct m6A patterns (m6Acluster.A and m6Acluster.B) based on the expression levels of all 21 m6A RNA methylation regulators from The Cancer Genome Atlas (TCGA) database using a consensus clustering algorithm. Differential analysis of m6Acluster.A and m6Acluster.B identified 196 m6A-related genes. We further validated the m6A regulation mechanism based on the 196 m6A-related genes using another consensus clustering algorithm. Considering individual differences, principal component analysis algorithms were used to calculate an m6A score for each sample in order to quantify the m6A patterns. A low m6A score was associated with immune activation and enhanced response to immune checkpoint inhibitors, whereas a high m6A score was associated with tumor progression. Finally, we successfully verified the correlation between m6A regulators and immune microenvironment in OC using our microarray analysis data. In summary, m6A regulators play non-negligible roles in immune infiltrates of ovarian cancer. Our investigation of m6A patterns may help to guide future immunotherapy strategies for advanced ovarian cancer.

## Introduction

Ovarian cancer is the deadliest gynecological malignancy because of its insidious onset and lack of effective early detection indicators (Siegel et al., 2018; Yang et al., 2020). The current standard treatment regimen involves ovarian tumor cell reduction plus platinum-based chemotherapy. However, most patients experience relapse within 12–24 months and die due to chemotherapy resistance (Chen et al., 2018; Maas et al., 2020). Therefore, it is critical to explore the pathogenesis of ovarian cancer and identify novel treatment strategies.

Methylation of the adenosine base at the N6 position, termed N6-methyladenosine (m6A), is an important epigenetic modification that requires the collective participation of multiple regulatory proteins (Hong, 2018). Regulators of m6A, the most abundant form of RNA modification, play significant roles in RNA processing, transportation, localization, translation, and degradation (Zhao et al., 2018). Numerous lines of evidence suggest that m6A regulators are involved in cell death, proliferation, immunomodulation, drug resistance, invasion, and tumor metastasis (Liu et al., 2020; Xu et al., 2020; Xue et al., 2020). Jiang et al. (2020) reported that the RNA demethylase ALKBH5 promotes the development of ovarian cancer in a simulated tumor microenvironment through stimulation of the nuclear factor-κB pathway. YTHDF2, an m6A mRNA reader inhibited by miR-145, plays a role in the proliferation, migration, and apoptosis of ovarian cancer cells (Li et al., 2020). YTHDF1 can upregulate the expression of TRIM29 to promote cisplatin resistance in ovarian cancer (Hao et al., 2021). However, there has been no comprehensive analysis of the relationship between m6A regulators and immune infiltrates of ovarian cancer.

Recent studies have shown that the tumor microenvironment (TME) is a key factor in tumor growth, metastasis, and regulation of the tumor immune response (Ngwa et al., 2019). Tumor immunotherapy implements the principle of immunology to reactivate and enhance the body’s anti-tumor immune response in specific ways, for example by using the immune system to eliminate tumor cells in order to suppress tumor growth. Immune checkpoint molecules, including CTLA-4, PD-1, TIM-3, LAG3, and TIGIT, are co-inhibitory molecules expressed on the surface of T cells that negatively regulate T cell signaling pathways and play an important role in inhibiting the anti-tumor immune activation of T cells (Velcheti and Schalper, 2016). Several immune checkpoint inhibitors (ICIs), such as Nivolumab, Pembrolizumab, Atezolizumab, Avelumab, and Durvalumab, have been approved by the United States. Food and Drug Administration for the treatment of advanced melanoma, non-small cell lung cancer, kidney cancer, stomach cancer, and liver cancer (Wang et al., 2019). However, there is limited experimental evidence to suggest that ovarian cancer benefits from ICIs used as single agents or in combination.

In this study, we aimed to comprehensively analyze the relationship between m6A regulators and immune infiltrates of ovarian cancer. The findings of this study may help to guide future therapeutic strategies for advanced ovarian cancer.

## Materials and Methods

### Data Acquisition

The transcriptome profiling datasets of 379 ovarian cancer samples, single-nucleotide variations of 436 ovarian cancer samples, and corresponding clinical information were downloaded from TCGA database^1^. The 21 m6A regulators included 8 writers (METTL3, ZC3H13, METTL14, RBM15B, CBLL1, WTAP, RBM15, and KIAA1429), 2 erasers (FTO and ALKBH5), and 11 readers (YTHDC1, YTHDC2, ELAVL1, YTHDF1, LRPPRC, YTHDF2, FMR1, YTHDF3, HNRNPC, HNRNPA2B1, and IGF2BP1) (Zhang et al., 2020).

### Specimen Collection

A total of 60 ovarian cancer samples were collected at ShengJing Hospital of China Medical University (Shenyang, China) from August 2019 to February 2021. The inclusion criteria for patients with ovarian cancer were: (1) High-grade serous ovarian cancer diagnosed by postoperative pathology; (2) Absence of chemotherapy, radiotherapy, immunotherapy, and other treatments before surgery; (3) No history of other tumors or ovary-related diseases. This study was approved by the ethics committee of the ShengJing Hospital of China Medical University, and informed consent was obtained from all patients. Then, mRNA expression microarray for the 60 ovarian cancer samples was performed in Kangcheng Biological (Shanghai, China) for validation analysis. The chip used Arraystar Human mRNA Microarray V4.0 (Arraystar, Rockville, MD, United States). The GeneSpring GX software V12.1 software (Agilent Technologies, Palo Alto, CA, United States) was used for quantile standardization of the original data.

### Consensus Clustering of the 21 m6A Regulators

We selected the gene expression data of the 21 m6A regulators from the transcriptome profiling datasets in order to identify distinct m6A patterns using a consensus clustering algorithm. The “ConsensusClusterPlus” package in R software (The R Foundation, Vienna, Austria) was used, and 1,000 repetitions were performed to ensure the stability of the classification (Wilkerson and Hayes, 2010).

### Consensus Clustering of the m6A-Related Genes

Differentially expressed genes between distinct m6A patterns were screened using the “limma” package in R. The genes with *P* < 0.01 and | log_2_ fold change ≥ 2| were considered significantly different in expression and categorized as m6A-related genes (Ritchie et al., 2015). Next, a consensus clustering algorithm was used to identify distinct m6A gene patterns based on the m6A-related genes, and 1,000 repetitions were performed to ensure the stability of the classification.

### Gene Ontology Functional Enrichment Analysis

Gene ontology (GO) analysis is a common method for annotating genes that can be used to identify gene enrichment in the biological process, cellular component, and molecular function categories. Along with the GO database, the enrichment function of m6A-related genes was analyzed and visualized using the “clusterProfiler” package in R. A significant enrichment with a statistically significant difference was determined based on the following conditions: a false discovery rate < 0.05 and adjusted *P* < 0.05 (Pomaznoy et al., 2018; Vinterhalter et al., 2020).

### Calculation of the m6A Score for Each Sample

Considering the individual differences, principal component analysis (PCA) algorithms were used to calculate an m6A score for each sample in order to quantify the m6A patterns. The PCA algorithm focuses on the largest set of highly related (or unrelated) gene blocks in the set and down-weights the contributions of those genes that are not tracked with other set members. According to a previous study (Zhang et al., 2020), we first distinguished the m6A gene patterns using PCA. Next, the m6A score was calculated according to the following formula: m6A score = Σ (PC1_*i*_ + PC2_*i*_), where PC1 represents principal component 1, PC2 represents principal component 2, and i represents the m6A-related genes.

### Statistical Analysis

All statistical analyses and drawings were performed using R version 4.0.0. We visualized the mutation data of ovarian cancer using the “maftools” package (Bi et al., 2020). The correlation coefficients between the 21 m6A RNA methylation regulators were calculated using Spearman correlation analyses. A univariate Cox regression model was utilized to evaluate the prognostic value of the 21 m6A regulators. Single sample gene set enrichment analysis was used to calculate the abundance of immune cells in ovarian cancer samples (Zhang et al., 2020). The tumor mutation burden (TMB) of each sample was calculated based on the following formula: TMB = Sn × 1,000,000/n, where Sn represents the absolute number of somatic mutations and n represents the number of exon bases covered at a depth ≥ 100 × (Bi et al., 2020). The Mann-Whitney test was performed to compare any differences between groups. All parametric analyses were based on two-tailed tests, for which the statistical significance was set at *P* < 0.05 (Hazra and Gogtay, 2016). Survival analysis was visualized using Kaplan-Meier curves.

## Results

### Landscape of the 21 m6A Regulators in Ovarian Cancer

**FIGURE 1:**
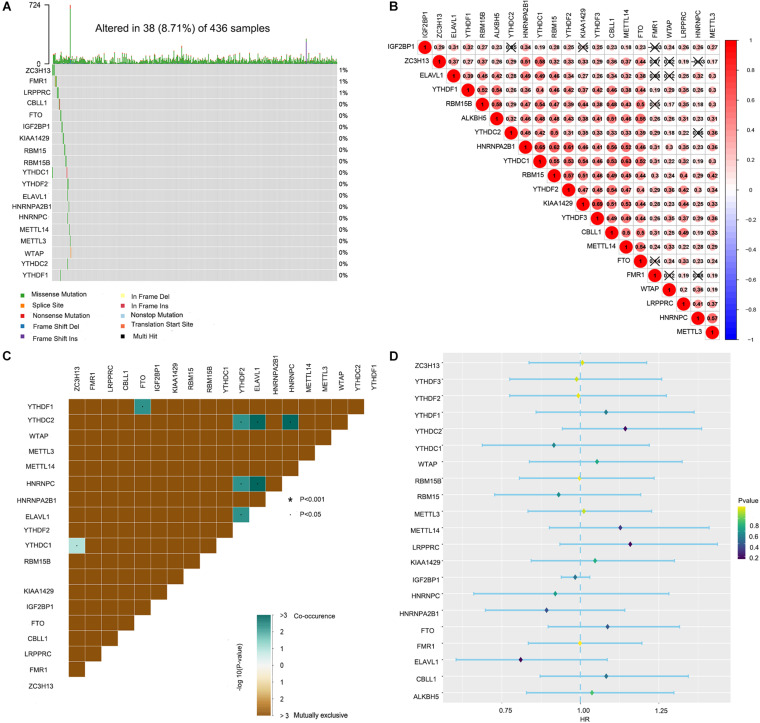
Landscape of the 21 RNA N6-methyladenosine (m6A) regulators in ovarian cancer from The Cancer Genome Atlas (TCGA) database. **(A)** The mutation characteristics of the 21 m6A regulators in 436 samples from TCGA-OV dataset. **(B)** The correlation coefficients of the 21 m6A regulators obtained by Pearson correlation analysis. **(C)** The mutation co-occurrence and exclusion analyses of the 21 m6A regulators. **(D)** The univariate Cox regression model used to evaluate the prognostic value of the 21 m6A regulators in ovarian cancer (*P < 0.05).

Waterfall plots were drawn to evaluate the somatic mutation incidence of the 21 m6A RNA methylation regulators in ovarian cancer samples from TCGA-OV dataset. The results revealed that among 436 ovarian cancer samples, only 38 samples had somatic mutations in the 21 m6A RNA methylation regulators. ZC3H13 displayed the highest mutation frequency among the 21 m6A regulators (Figure 1A). The OV samples in single-nucleotide variation datasets did not include samples with ALKBH5 and YTHDF3 mutations. The mutation frequency of METTL3, METTL14, RBM15B, CBLL1, WTAP, RBM15, KIAA1429, FTO, YTHDC1, YTHDC2, ELAVL1, YTHDF1, YTHDF2, HNRNPC, HNRNPA2B1, and IGF2BP1 in the OV samples was close to 0%. Spearman correlation analyses were utilized to calculate the correlation coefficients between the 21 m6A regulators. We found that KIAA1429 had the highest positive correlation with YTHDF3 (correlation coefficient: 0.69; Figure 1B). The mutation co-occurrence and exclusion analyses of the 21 m6A regulators using the “maftools” package in R revealed that ELAVL1, HNRNPC, and YTHDC2 had the highest mutation co-occurrence association (Figure 1C). Unfortunately, the univariate Cox regression model showed that the 21 m6A regulators had no significant prognostic value in ovarian cancer (Figure 1D).

### Construction of Two Distinct m6A Patterns Based on the 21 m6A Regulators

**FIGURE 2:**
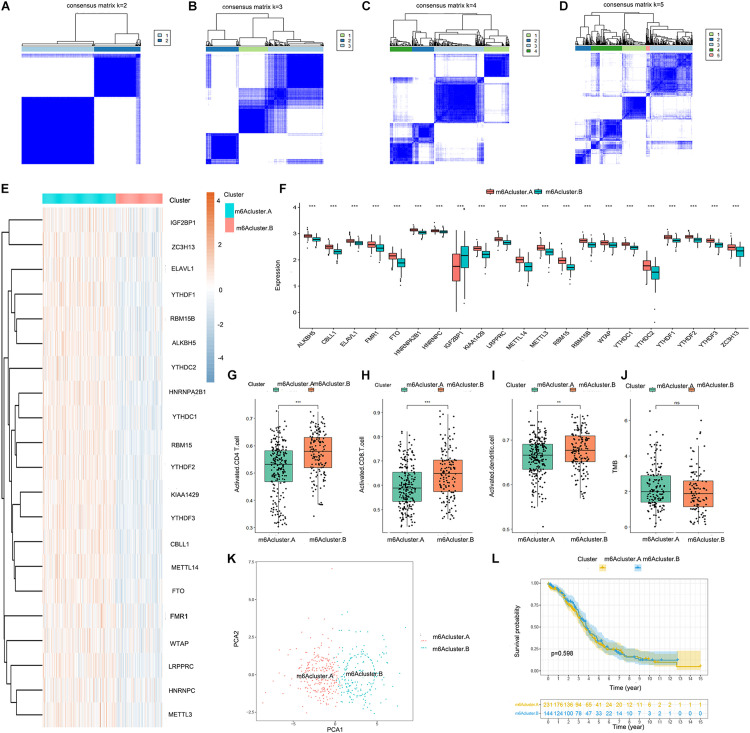
Consensus clustering of the 21 m6A regulators in TCGA-OV dataset. **(A–D)** Consensus matrices of the 21 m6A regulators for *k* = 2–5. **(E)** Expression heat map of the 21 m6A regulators in m6Acluster.A and m6Acluster.B. **(F)** Differential expression boxplot of the 21 m6A regulators in m6Acluster.A and m6Acluster.B. Differences in the abundance of activated CD4 T cell **(G)**, CD8 T cell **(H)**, and dendritic cell **(I)** infiltrates between m6Acluster.A and m6Acluster.B. **(J)** Differences in the tumor mutation burden (TMB) level between m6Acluster.A and m6Acluster.B. **(K)** Principal component analysis (PCA) for the expression profiles of the 21 m6A regulators, showing a remarkable difference in transcriptomes between the 2 m6A patterns. The variance of PCA1 was 39.67, while the variance of PCA2 was 19.92. **(L)** Kaplan-Meier curves showing the prognostic value of the two m6A patterns in TCGA-OV dataset (**P < 0.01 and ***P < 0.001).

We obtained two distinct m6A patterns (m6Acluster.A and m6Acluster.B) based on the expression levels of the 21 m6A regulators using a consensus clustering algorithm (Figures 2A–D). The heat map and boxplot revealed that the expression of all 21 m6A regulators except IGF2BP1 was higher in m6Acluster.A than in m6Acluster.B (Figures 2E,F). Principal component analysis indicated that the 21 m6A regulators could effectively distinguish between patients in m6Acluster.A and m6Acluster.B (Figure 2K). We also found that the abundance of activated CD4 T cells, activated CD8 T cells, and activated dendritic cells was higher in patients in m6Acluster.B than in patients in m6Acluster.A (Figures 2G–I). However, there were no significant differences in prognosis (Figure 2L) and TMB level (Figure 2J) between m6Acluster.A and m6Acluster.B.

### Establishment of Two Distinct m6A Gene Patterns Based on the m6A-Related Genes

**FIGURE 3:**
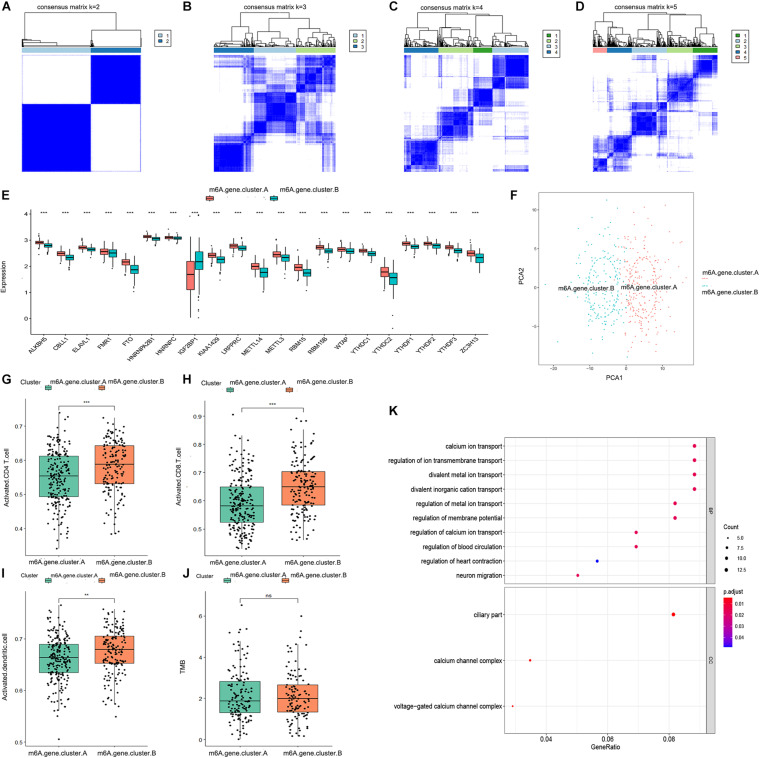
Consensus clustering of the 196 m6A-related genes in TCGA-OV dataset. **(A–D)** Consensus matrices of the 196 m6A-related genes for *k* = 2–5. **(E)** Differential expression boxplot of the 21 m6A regulators in m6A.gene.cluster.A and m6A.gene.cluster.B. **(F)** PCA for the expression profiles of the 196 m6A-related genes, showing a remarkable difference in transcriptomes between the two m6A gene patterns. The variance of PCA1 was 45.23, while the variance of PCA2 was 14.94. Differences in the abundance of activated CD4 T cell **(G)**, CD8 T cell **(H)**, and dendritic cell **(I)** infiltrates between m6A.gene.cluster.A and m6A.gene.cluster.B. **(J)** Differences in the TMB level between m6A.gene.cluster.A and m6A.gene.cluster.B. **(K)** Gene ontology (GO) analysis exploring the potential mechanisms underlying the effect of the 196 m6A-related genes on the occurrence and development of ovarian cancer. “Count” represents “number of enriched genes.” “GeneRatio” represents “number of enriched genes/number of total genes.” The size of the dots represents the number of genes enriched, and the color represents p. adjust. The redder the color, the smaller the p. adjust (**P < 0.01 and ***P < 0.001).

We obtained 196 m6A-related genes between the two distinct m6A patterns using the “limma” package in R. Consistent with the two distinct m6A patterns, we also found two distinct m6A gene patterns (m6A.gene.cluster.A and m6A.gene.cluster.B) based on the 196 m6A-related genes using a consensus clustering algorithm (Figures 3A–D). The boxplot indicated that the expression of all 21 m6A regulators except IGF2BP1 was higher in m6A.gene.cluster.A than in m6A.gene.cluster.B, which was consistent with the expression in the two distinct m6A patterns (Figure 3E). PCA also indicated that the 196 m6A-related genes could effectively distinguish between patients in m6A.gene.cluster.A and m6A.gene.cluster.B (Figure 3F). Activated CD4 T cells, activated CD8 T cells, and activated dendritic cells were more abundant in m6A.gene.cluster.B than in m6A.gene.cluster.A (Figures 3G–I). There was no significant difference in the TMB level between m6A.gene.cluster.A and m6A.gene.cluster.B (Figure 3J). Collectively, the results demonstrated strong stability of the consensus clustering algorithm in sample classification. Finally, GO analysis was used to reveal the possible mechanisms by which the 196 m6A-related genes affect the progression of ovarian cancer. As shown in Figure 3K, the 196 m6A-related genes were primarily enriched in regulation of calcium ion transport, calcium ion transport, regulation of metal ion transport, and regulation of blood circulation, all of which are associated with signal transduction.

### Significance of the m6A Score in Ovarian Cancer

**FIGURE 4:**
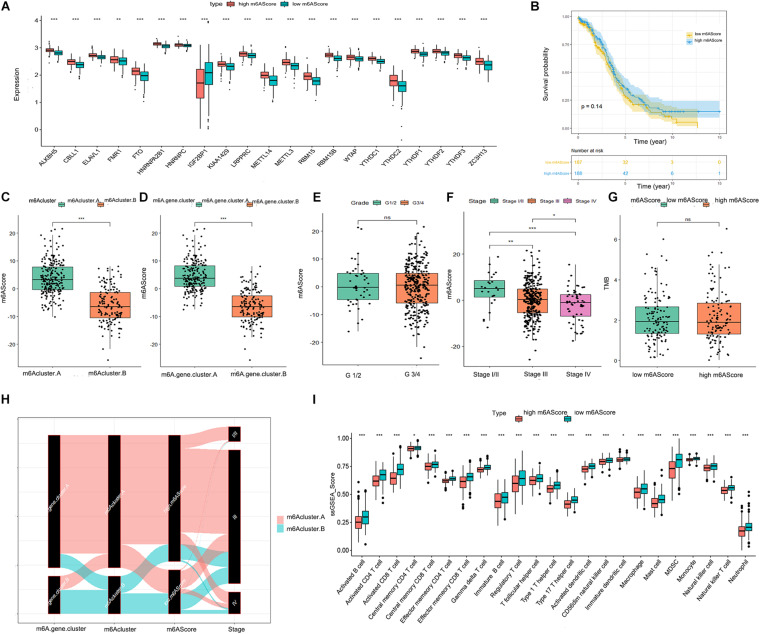
Significance of the m6A score in ovarian cancer. **(A)** Differential expression boxplot of the 21 m6A regulators in high and low m6A scores. **(B)** Kaplan-Meier curves showing the prognostic value of the m6A score in TCGA-OV dataset. Differences in m6A score between m6Acluster.A and m6Acluster.B **(C)** and m6A.gene.cluster.A and m6A.gene.cluster.B **(D)**. **(E)** Differences in m6A score between tumor grade (G)1/2 and G3/4. **(F)** Differences in m6A score between different stages of ovarian cancer. **(G)** Differences in the TMB level between high and low m6A scores. **(H)** Sankey diagram revealing the relationship between m6Acluster, m6A.gene.cluster, m6A scores, and stage. The abscissa represents the groups. Black boxes represent the grouping of each group. Red represents the patients in the m6Acluster.A group, while green represents the patients in the m6Acluster.B group. **(I)** Differential immune cell infiltration between high and low m6A scores (*P < 0.05, **P < 0.01, and ***P < 0.001).

To quantify the m6A patterns, PCA algorithms were performed to calculate an m6A score for each ovarian cancer sample. The boxplot indicated that the expression of all 21 m6A regulators except IGF2BP1 was higher in the high m6A score group than in the low m6A score group (Figure 4A). The patients in m6Acluster.A and m6A.gene.cluster.A had higher m6A scores than those in m6Acluster.B and m6A.gene.cluster.B (Figures 4C,D). As illustrated in Figure 4F, there was a negative correlation between advanced ovarian cancer stage and m6A score. However, the m6A score had no statistical correlation with prognosis, TMB level, or pathological grading of ovarian cancer (Figures 4B,E,G). From the above results, we inferred that there are complex correlations among m6Acluster, m6A.gene.cluster, m6A scores, and stage. To better understand these correlations, we drew a Sankey diagram (Figure 4H). Finally, as shown in Figure 4I, patients with a lower m6A score had a higher abundance of infiltrating immune cells.

### Biological Phenotypes Associated With the m6A Score in Ovarian Cancer

**FIGURE 5:**
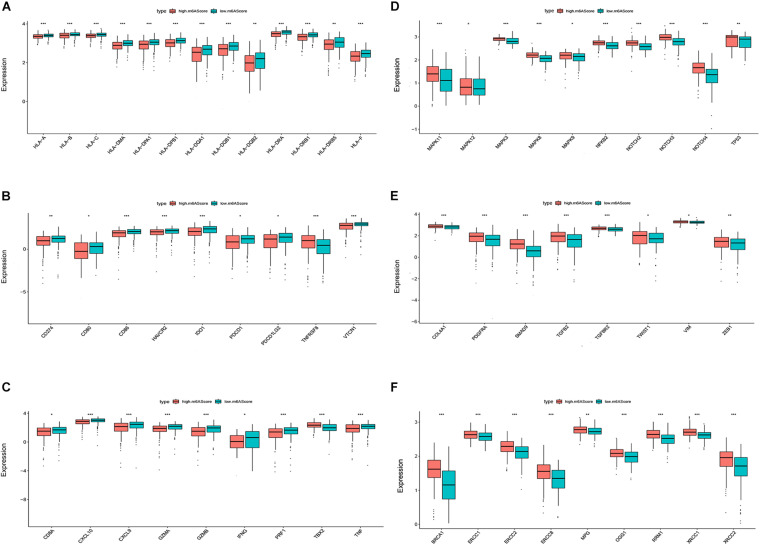
Biological phenotypes associated with the m6A score in ovarian cancer. Differential expression of human leukocyte antigen (HLA) genes **(A)**, immune checkpoint molecules **(B)**, immune activation-related genes **(C)**, proliferation-related genes **(D)**, DNA repair-related genes **(E)**, and transforming growth factor (TGF)β-epithelial mesenchymal transition (EMT) pathway-related genes **(F)** between the high and low m6A score groups (*P < 0.05, **P < 0.01, and ***P < 0.001).

We then investigated the biological phenotypes related to the m6A score in ovarian cancer. The results indicated that human leukocyte antigen (HLA) genes, immune checkpoint molecules, and immune activation-related genes were more highly expressed in the low m6A score group than in the high m6A score group, suggesting that patients with a low m6A score may be more sensitive to ICIs (Figures 5A–C). The expression levels of proliferation-related and DNA repair-related genes [1, 2] were higher in the high m6A score group than in the low m6A score group, suggesting that a high m6A score is likely to be associated with poor ovarian cancer prognosis (Figure 5D–F).

### Differences in Genetic Mutations Between Groups With High and Low m6A Scores

**FIGURE 6:**
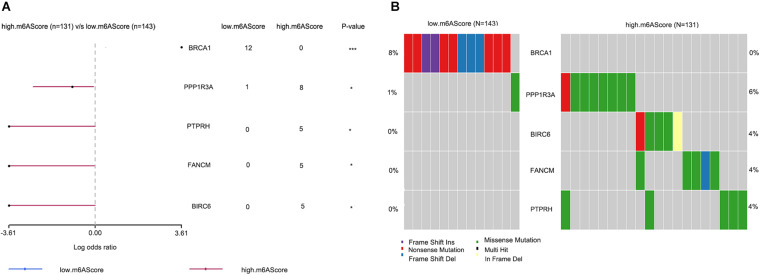
Differences in genetic mutations between the high and low m6A score groups. **(A)** Forest plots showing five differentially mutated genes between the high and low m6A score groups. **(B)** Co-onco plots showing five differentially mutated genes between the high and low m6A score groups (*P < 0.05 and ***P < 0.001).

To reveal the differential gene mutations between groups with high and low m6A scores, the “maftools” package was used, and the results were visualized using forest plots (Figure 6A) and co-onco plots (Figure 6B). The OV samples in transcriptome profiling datasets were not consistent with the OV samples in single-nucleotide variation datasets. Thus, we only obtained 274 overlapping samples showing differences in genetic mutations. We found five differentially mutated genes (*BRCA1, PPP1R3A, PTPRH, FANCM*, and *BIRC6*) between the groups with high and low m6A scores. Aside from that of *BRCA1*, the mutation rates of the remaining genes were higher in the high m6A score group than in the low m6A score group.

### Validation of the Correlation Between m6A Regulators and Immune Microenvironment in Ovarian Cancer

**FIGURE 7:**
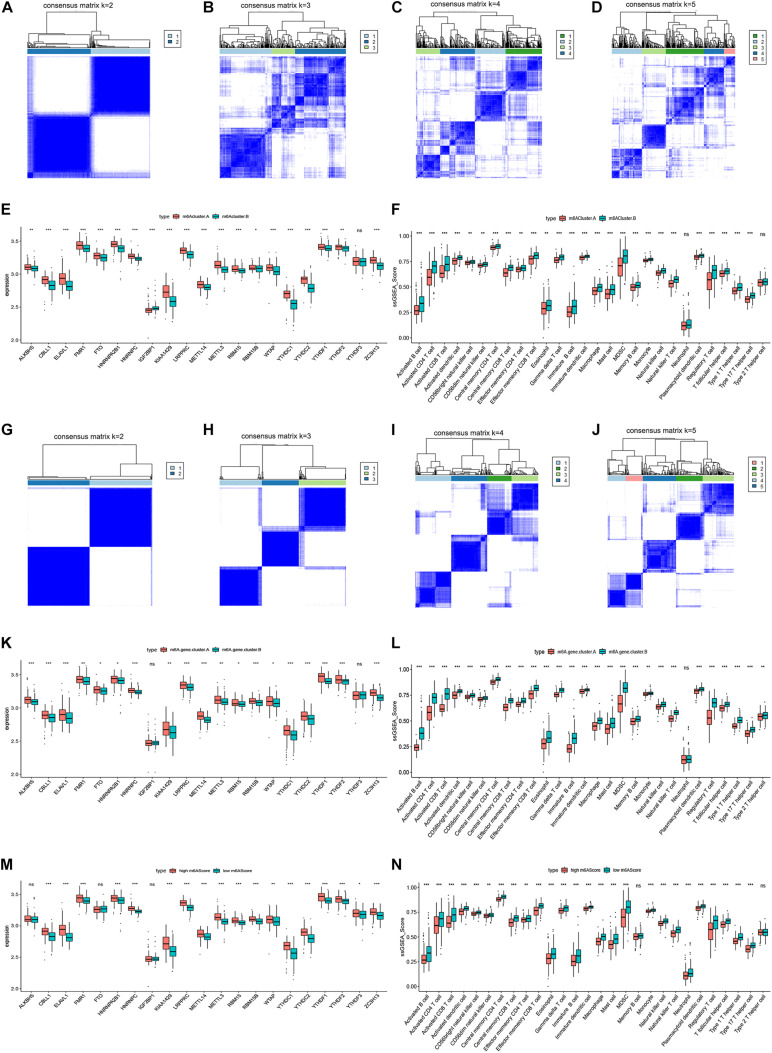
Validation of the correlation between m6A regulators and immune microenvironment in ovarian cancer. **(A–D)** Consensus matrices of the 21 m6A regulators for *k* = 2–5. **(E)** Differential expression boxplot of the 21 m6A regulators in m6Acluster.A and m6Acluster.B. **(F)** Differential immune cell infiltration between m6Acluster.A and m6Acluster.B. **(G–J)** Consensus matrices of the 125 m6A-related genes for *k* = 2–5. **(K)** Differential expression boxplot of the 21 m6A regulators in m6A.gene.cluster.A and m6A.gene.cluster.B. **(L)** Differential immune cell infiltration between m6A.gene.cluster.A and m6A.gene.cluster.B. **(M)** Differential expression boxplot of the 21 m6A regulators in high and low m6A scores. **(N)** Differential immune cell infiltration between high and low m6A scores (*P < 0.05, **P < 0.01, and ***P < 0.001).

To verify our results, we performed microarray sequencing on 60 ovarian cancer samples collected at ShengJing Hospital of China Medical University. The 21 m6A regulators were extracted and the consensus clustering algorithm was used. Consistent with the results of the original analysis, ovarian cancer patients were divided into two groups (m6Acluster.A and m6Acluster.B, Figures 7A–D). The boxplot indicated that the expression of all 21 m6A regulators except IGF2BP1 and YTHDF3 was higher in the m6Acluster.A group than in the m6Acluster.B group (Figure 7E). In addition, patients in the m6Acluster.B group showed increased immune infiltration compared with patients in the m6Acluster.A group (Figure 7F). Moreover, 125 m6A-related genes were acquired between the two distinct m6A patterns based on a differential expression analysis according to the screening criteria *p* < 0.05 and | log_2_ fold change ≥ 2|. We then obtained two distinct m6A gene patterns (m6A.gene.cluster.A and m6A.gene.cluster.B) based on the 125 m6A-related genes using a consensus clustering algorithm (Figures 7G–J). The boxplot indicated that the expression of all 21 m6A regulators except IGF2BP1 and YTHDF3 was higher in the m6A.gene.cluster.A group than in the m6A.gene.cluster.B group (Figure 7K). In addition, patients in the m6A.gene.cluster.B group showed higher immune infiltration (Figure 7L). Finally, PCA algorithms were used to calculate an m6A score for each ovarian cancer sample and quantify the m6A patterns. As expected, expression of most m6A regulators was higher in the high m6A score group (Figure 7M), and the patients in the low m6A score group had a higher abundance of infiltrating immune cells (Figure 7N). Together, these results confirmed that the m6A regulators have a stable relationship with the immune microenvironment in ovarian cancer.

## Discussion

The immune system is an important barrier to the occurrence and development of malignant tumors; adaptive T cell immunity exerts the most important anti-tumor effect (Yang, 2015). However, the abundance of infiltrating T cells in the TME is low owing to the low immunogenicity of solid tumors, which mostly originate from mutations in epithelial or stromal cells (Khalil et al., 2015). In addition, tumor cells can use a variety of mechanisms, including recruitment of inhibitory cells, production of inhibitory cytokines and chemokines, expression of inhibitory molecules, and metabolic competition, to limit the infiltration of T cells (Anderson et al., 2017). Therefore, improving the infiltration and function of tumor-specific T cells is one of the important strategies of anti-tumor immunotherapy. Numerous lines of evidence have revealed that m6A regulators play an indispensable role in tumor immunity. However, the relationship between m6A regulators and immune infiltrates of ovarian cancer remains unexplored. The aim of this research was to comprehensively analyze the role of m6A regulators in anti-tumor immunity and provide guidance for the immunotherapy of ovarian cancer.

In the present study, we obtained two distinct m6A patterns based on the expression levels of 21 m6A regulators. Patients in the m6Acluster.B group showed a higher abundance of activated CD4 T cells, activated CD8 T cells, and activated dendritic cells than patients in the m6Acluster.A group, indicating that the m6Acluster.B is related to elevated immune activity. Further, we obtained 196 m6A-related genes through differential analysis of the two distinct m6A patterns. Surprisingly, we found two distinct m6A gene patterns based on the expression levels of the 196 m6A-related genes that were similar to the m6A patterns. Activated CD4 T cells, activated CD8 T cells, and activated dendritic cells were more abundant in the m6A.gene.cluster.B, and the 196 m6A-related genes were mainly enriched in immune activity-related biological functions. These observations revealed that the consensus clustering algorithm has strong stability in sample classification and that m6A regulators have great significance in immune infiltrates of ovarian cancer. Considering the individual differences, PCA algorithms were performed to calculate an m6A score for each sample in order to quantify the m6A patterns. Sankey diagram showed that the patients in m6Acluster.A and m6A.gene.cluster.A had higher m6A scores than those in m6Acluster.B and m6A.gene.cluster.B. The patients with advanced ovarian cancer stage have lower m6A score, suggesting that the m6A regulators play any role in ovarian tumor aggressiveness. We also found that the m6A patterns characterized by elevated immune activity exhibited a lower m6A score. In addition, patients with a low m6A score showed higher expression levels of HLA genes, immune checkpoint molecules, and immune activation-related genes, consistent with our expectations. Together, these results indicate that patients with lower m6A scores may be more sensitive to ICIs. Since a lower m6A score was associated with an advanced pathological stage of ovarian cancer, we speculated that advanced ovarian cancer patients may benefit from ICIs. However, the m6A score showed no statistical correlation with ovarian cancer prognosis. Thus, we suggest that patients who are highly sensitive to ICIs can only improve their survival time by receiving ICI therapy. Moreover, we successfully verified the correlation between m6A regulators and the immune microenvironment in ovarian cancer using our microarray analysis data, which greatly adds to the credibility of our study. Our data also indicated that the m6A score is negatively correlated with proliferation, the transforming growth factor (TGF)β-epithelial mesenchymal transition (EMT) pathway, and DNA damage repair of ovarian cancer. These observations suggest that lower m6A scores and sensitivity to ICIs are not only related to elevated immune activity and overexpression of immune checkpoint molecules but also associated with the suppression of proliferation, the TGFβ-EMT pathway, and DNA damage repair. Consistent with our results, previous studies have revealed that the TGFβ pathway is associated with immune evasion of tumors by restricting T cells (Mariathasan et al., 2018; Tauriello et al., 2018).

Finally, we revealed five differentially mutated genes (*BRCA1, PPP1R3A, PTPRH, FANCM*, and *BIRC6*) between the high and low m6A score groups. Aside from that of *BRCA1*, the mutation rates of the remaining genes were higher in the high m6A score group than in the low m6A score group. Previous studies have indicated that ovarian cancer subtypes with higher *BRCA1* mutations correspond to environments with higher expression levels of immune checkpoint molecules and elevated levels of infiltrating immune cells, consistent with our results (Wei et al., 2020; Zheng et al., 2020). However, there are no studies on the relationship between the other four mutated genes and ovarian cancer immunity. We will utilize this knowledge gap as a research direction for further experimental studies and ideally provide novel insights into the treatment of ovarian cancer.

## Conclusion

In conclusion, our research identified two distinct m6A patterns based on 21 m6A regulators and calculated an m6A score for each sample to quantify the m6A patterns using PCA algorithms. A low m6A score represented more sensitivity to ICIs. These findings may help develop a potential therapeutic strategy for advanced ovarian cancer.

## Data Availability Statement

The original contributions presented in the study are included in the article/Supplementary Material, further inquiries can be directed to the corresponding author/s.

## Author Contributions

JG and FB conceived and designed the study, developed the methodology, analyzed and interpreted the data, and wrote, reviewed, and/or revised the manuscript. Both authors contributed to the article and approved the submitted version.

## Conflict of Interest

The authors declare that the research was conducted in the absence of any commercial or financial relationships that could be construed as a potential conflict of interest.

## Footnotes

1 https://portal.gdc.cancer.gov/
