# Supplementary figures and images for: Significance of N6-Methyladenosine RNA Methylation Regulators in Immune Infiltrates of Ovarian Cancer
Source: Front Genet. 2021 Jul 7;12:671179. doi: 10.3389/fgene.2021.671179 (PMC8295008; doi:10.3389/fgene.2021.671179)

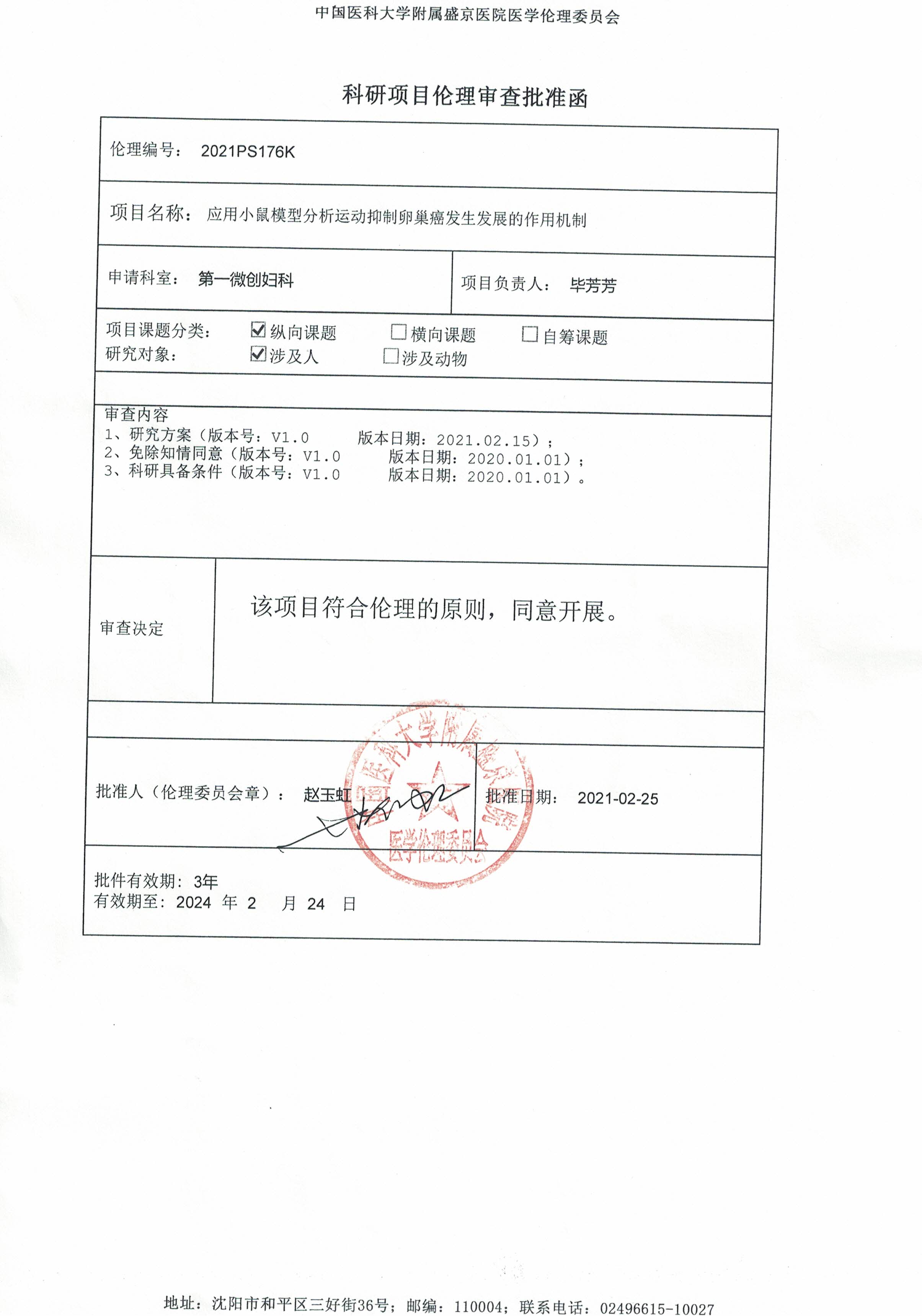

Supplement: Supplementary file 1 [file Image_1.JPEG]
